# The Latent Structure of Autistic Traits: A Taxometric, Latent Class and Latent Profile Analysis of the Adult Autism Spectrum Quotient

**DOI:** 10.1007/s10803-016-2897-z

**Published:** 2016-09-12

**Authors:** Richard J. E. James, Indu Dubey, Danielle Smith, Danielle Ropar, Richard J. Tunney

**Affiliations:** School of Psychology, University Park, University of Nottingham, Nottingham, NG7 2RD UK

**Keywords:** Autism quotient, Autistic traits, Taxometric analysis, Latent class analysis, Latent structure analysis, Nosology

## Abstract

Autistic traits are widely thought to operate along a continuum. A taxometric analysis of Adult Autism Spectrum Quotient data was conducted to test this assumption, finding little support but identifying a high severity taxon. To understand this further, latent class and latent profile models were estimated that indicated the presence of six distinct subtypes: one with little probability of endorsing any autistic traits, one engaging in ‘systemising’ behaviours, three groups endorsing multiple components of Wing and Gould’s autistic triad, and a group similar in size and profile to the taxon previously identified. These analyses suggest the AQ (and potentially by extension autistic traits) have a categorical structure. These findings have important implications for the analysis and interpretation of AQ data.

**Electronic supplementary material:**

The online version of this article (doi:10.1007/s10803-016-2897-z) contains supplementary material, which is available to authorized users.

## Introduction

 Research suggests that autistic traits (AT) may be higher in first degree relatives of people with Autism Spectrum Disorder (ASD), even though they might not meet the criteria for clinical diagnosis (Hoekstra et al. 2007a, b). These behaviours potentially represent a broader phenotype of autism (Hoekstra et al. 2007a) that may be valuable in understanding the behavioural and cognitive profile of people with ASD. ASD in the DSM-5 refers to a dyad of impairments in restricted, repetitive behaviours (RRBs), and social communication and interaction (American Psychiatric Association 2013). The possibility that there is a range of behaviours that differ by degree in the population implies a dimensional approach to ASD rather than a simple categorization of individuals, with ASD representing the extreme tail of a distribution. It has further been claimed that ATs are continuously distributed amongst the population, and that discontinuities only emerge with comorbid learning difficulties for a subsample of those with ASD (Ruzich et al. 2015). This may provide insights into the underlying causal mechanisms behind ASD (Happe et al. 2006).

This approach has led to the increasing use of tools to measure ATs. One of the most frequently used measures is the Adult Autism Spectrum Quotient (AQ) (Baron-Cohen et al. 2001). The AQ is a 50 item, self-report measure in which participants are asked to rate their agreement to an item on a 4-point scale that is subsequently dichotomised. The questionnaire yields an overall score that is intended to quantitatively represent the severity of AT. The scale was intended to measure five subscales of AT: social skills, attention switching, attention to detail, communication, and imagination (Baron-Cohen et al. 2001). Although the AQ ostensibly measures AT, researchers in the field have also referred to the scale measuring ‘autistic like traits’ (or ALT (Happe et al. 2006; Ronald et al. 2006; Lundström et al. 2012)), possibly representing the apprehension concerning whether autistic traits directly correspond to clinical symptoms of ASD. This highlights the need to clarify the nature of the characteristics as measured by the AQ.

Exploratory factor analyses of AQ data have demonstrated divergent findings on the dimensional structure of the AQ suggesting the presence of two (Hoekstra et al. 2008), three (Hurst et al. 2007; Austin 2005; Palmer et al. 2015), four (Stewart and Austin 2009), or five factors (Kloosterman et al. 2011). Though these studies differ from one another in the suggested number of factors, they all identify social skills and attention to detail/patterns as major components measured by the AQ. The AQ has been found to have strong internal reliability (α = 0.82) (Austin 2005), good test–retest reliability (ICC = 0.7) (Baron-Cohen et al. 2001), and has demonstrated high internal validity across different cultures (Woodbury-Smith et al. 2005; Broadbent et al. 2013; Wakabayashi et al. 2006b). At a cut-off score of 26 for clinical samples, the AQ has high sensitivity (0.95) and specificity (0.52) in identifying individuals who have been clinically diagnosed with ASD (Woodbury-Smith et al. 2005). A cut off of 32 is recommended for non-clinical samples (Baron-Cohen et al. 2001; Woodbury-Smith et al. 2005). Studies suggest that the AQ can be a useful screening tool for discriminating ASD from a number of other psychopathologies (Wouters and Spek 2011; Cath et al. 2008; Sizoo et al. 2009). Due to its reported advantageous psychometric properties, researchers frequently use the AQ to measure the severity of ATs to predict performance of people with ASD (Miu et al. 2012; Rhodes et al. 2013). However no study to date has used the most robust statistical approaches to test whether respondents above or below a cutoff on the AQ, identified by the literature or modelling, primarily differ quantitatively (i.e. AQ score) or qualitatively (e.g. different subtypes endorsing different behaviours).

Previous studies (Palmer et al. 2015; Ring et al. 2008) have tested whether AQ data are continuous or discontinuous in nonclinical populations by identifying clusters that diverged from one another in terms of profile or severity of ATs endorsed respectively. However, the use of cluster or factor analysis is problematic in determining whether a latent construct is categorical or dimensional. Factor analysis assumes the presence of latent dimensions whereas cluster analysis does not reliably discriminate whether different clusters identify qualitatively distinct populations (Ruscio and Ruscio 2000), and performs poorly in Monte Carlo analyses compared to other approaches (Cleland et al. 2000). Findings from cluster analyses also tend to be difficult to replicate.

Consequently we report the first taxometric analysis of AQ data designed to address this question. Taxometric analysis is a method that tests whether differences between individuals on a latent construct are primarily quantitative or qualitative. Taxometric analyses have been used to study the latent structure of a wide range of self-completed (e.g. BDI, MMPI) and clinician administered assessments (ADI-R, DSM-IV SCID) across many different types of psychiatric disorder (Haslam et al. 2012). These findings can have implications on how the data from these tools should be analysed and interpreted. In this study our primary aim was to capture a sufficiently wide range of AQ scores (rather than ASD status) in order to study the psychometric properties of the AQ. In doing so we report the first taxometric and latent class analyses conducted on the AQ.

## Study 1: Taxometric Analysis of AQ Data

Taxometric analysis is a statistical approach designed to test whether a latent variable, measured by a number of ordinal or continuous observed variables, is categorical or continuous. Studies have demonstrated that taxometric analysis is better at discriminating latent structure relative to other psychometric techniques (McGrath and Walters 2012). Haslam et al. (2012), in reviewing the literature, found that the overwhelming majority of psychopathologies show a dimensional latent structure. However, three types of disorders: addictions, schizotypy and ASD were identified as potentially yielding taxa.

In taxometrics cases are assigned or not to a putative latent class, or taxon, on the basis of a cut-off, diagnosis, or base rate. Cases are then ordered along one of the indicators (the *input*), dividing them into ‘windows’ or ‘cuts’ and a statistical operation is performed on another variable/couplets of variables/remaining indicators (the *output*). Different taxometric procedures provide non-redundant information on the latent structure of the variable of interest (Ruscio et al. 2006). Plotting the output of taxometric analysis may reveal discontinuities that suggest a taxon, typically represented by a distinct peak. This however varies by levels of indicator validity, nuisance covariance, skew, kurtosis etc. Interpretation of taxometric findings typically include comparisons of bootstrapped datasets with idealised categorical and dimensional structures and comparing the disparity between the idealised and actual data to provide a quantitative index of fit between the two competing models (Haslam et al. 2012).

Previous taxometric analyses carried out on groupings of indicators derived from assessments (ADI-R, VABS, Peabody Picture Vocabulary test and the Raven Progressive Matrices) of ASD or characteristics associated with ASD found a number of quantitative and qualitative differences (Ingram et al. 2008). Categorical differences in social interaction, physical dysmorphologies and IQ were observed, and quantitative differences in ASD-related indicators such as insistence of sameness, repetitive motor actions, language acquisition and weaker evidence within adaptive functioning. A further study employing taxometric techniques has looked at questionnaire data in children (Frazier et al. 2010), using the Social Communication Questionnaire (Rutter et al. 2003) and Social Responsiveness Scale (Constantino and Gruber 2005). This found strong evidence using both taxometric and latent mixture modelling for a two-class taxonic model of ASD in children.

The continuity of ATs has been frequently assumed but has not been directly tested. A recent systematic review (Ruzich et al. 2015) and psychometric analysis of AQ data (Murray et al. 2014) have suggested that taxometric and latent class modelling would be beneficial. Previous taxometric analyses of ASD related constructs have found that a unimodal distribution of observed ATs need not necessarily correspond with a continuous latent structure (Frazier et al. 2010). It has been previously noted even when values appear to be normally distributed this may not entail a single population (Murphy 1964), indicating that a more sensitive analysis is warranted. While there is evidence to suggest that overall AQ scores might be continuous and the AQ is explicitly expected to measure multiple domains within this dimension (Baron-Cohen et al. 2001), taxometric analyses of other AT measures across the entire spectrum observed discontinuities.

## Method

### Sample

1139 cases were analysed from a sample of 1142 responses to the AQ collected from two separate studies. Respondents were sampled from the student community (58 % of sample) and online.

The first study (*n* = 619, 54.1 %) sampled adults online. 369 respondents were female, 227 male, and six identified with an alternative gender. Ages ranged from 16 to 70 (*M* = 26.46, *SD* = 10.00, median = 23). Participants were sampled from Reddit (*n* = 311, 27.3 %), social media (*n* = 193, 16.9 %), and an internal recruitment system for undergraduate students at the University of Nottingham for partial completion of course credit (*n* = 146, 12.8 %). The questionnaire was advertised on Reddit in areas relating to research in general, Asperger’s Syndrome and autism. Specific details of the number of respondents from each of the sub-forums sampled were not taken. Both of the ASD-related forums are aimed towards people with autism related conditions and their families and friends, and include discussion of autism related research. This means within the sample we are likely to have a small number of ASD cases, but the exact number is unknown. However, as the aim is to study the psychometric structure of the AQ, this does not detract from the purpose of this study. A sample ideal for taxometric analysis is likely to substantially deviate from the distribution of autistic traits in the general population.

A further 523 (45.9 %) participants (302 females; 221 males) were recruited primarily from the university community for a laboratory study. Participants’ ages ranged from 17 to 47 (*M* = 21.42, *SD* = 4.38, median = 20).

Across the entire sample, the mean AQ score was 21.52 (*SD* = 9.47, Range = 2–49, Median = 20) and was slightly, albeit significantly (*t*(1070.228) = 5.74, *p* < 0.001), higher in the online (*M* = 22.93, *SD* = 10.90, Range = 2–49, Median = 21) than the laboratory collected sample (*M* = 19.85, *SD* = 7.09, Range = 4–44, Median = 19). Although slightly higher than other AQ samples, there is fluctuation in AQ scores between samples in the literature (Ruzich et al. 2015), and ours is similar to other studies administering the AQ online (Palmer et al. 2015).

**Fig. 1:**
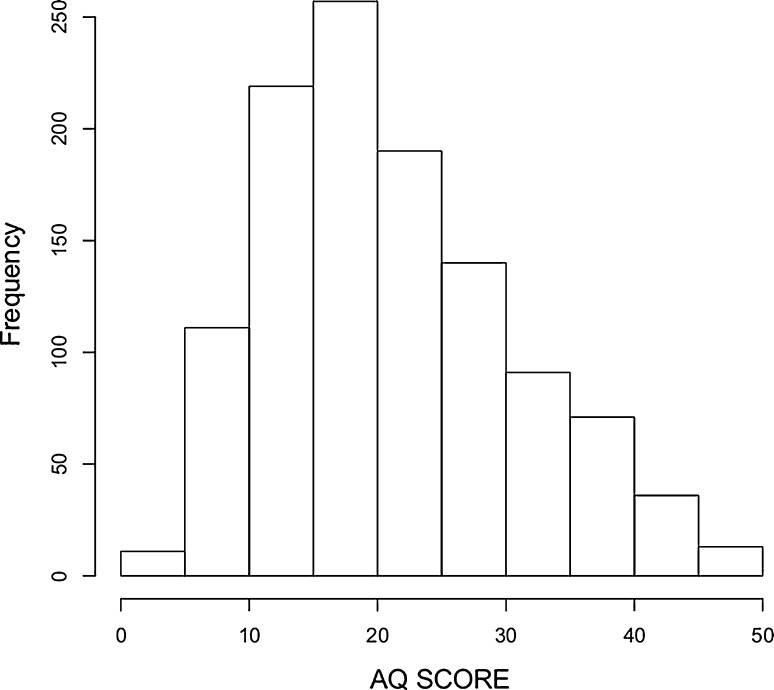
Histogram of AQ scores from both samples

The distribution of AQ scores was slightly positively skewed (γ = 0.566) and platykurtic (*b*_2_ − 3 = −0.32) and deviated from normality (Shapiro-Wilks test, *p* < 0.001). However, levels of skew and kurtosis did not differ substantially from other studies using the AQ (Ingersoll et al. 2011; Ujiie and Wakabayashi 2015; Wakabayashi et al. 2006a). Neither taxometric nor latent class analysis assumes normality so this does not preclude further analysis. Although it is assumed the AQ is normally distributed, the literature reporting distributions for the AQ is mixed, with some finding a normal distribution (Broadbent et al. 2013; Hurst et al. 2007), some reporting non-normal distributions (Murray et al. 2014; Puzzo et al. 2009), and many not reporting distributional statistics (Ruzich et al. 2015). A histogram of the distribution appears to follow the same broad pattern as reported in the systematic review of AQ data in non-clinical samples (Ruzich et al. 2015) (Fig. 1).

Cases with significant missing data (*n* = 3) were excluded from analysis. For two of these cases, only one question was completed. For the third, 19 responses were missing. 114 respondents had data missing from the online sample. For 90 % of these respondents fewer than five AQ items were missing. The number of items that an individual did not respond to ranged from 0 to 13.^1^ The level of missing data was small, and did not exceed 2.5 % for any single AQ item. Missing data were imputed rather than excluding cases with missing data from the analysis. Specific details regarding imputation are reported in the Supplementary Materials.

Ethical clearance was received from the University of Nottingham, School of Psychology Ethics Review Committee for data collection for both samples and for the present secondary analysis.

### Indicator Construction

Indicators were constructed from the AQ by conducting an exploratory factor analysis on the dataset to form subscales. Previous analyses have disagreed on the factor structure of the AQ and uses of empirically sound methods of retaining factors have produced spurious results (Stewart and Austin 2009). These factors are often highly correlated with one another and so may not be appropriate for taxometric analysis.

Factor retention was judged using multiple criteria. Parallel analysis was conducted using the *nFactors* package (Raiche and Magis 2015; Raiche et al. 2006). A comparison data approach, similar to the way taxometric plots are supplemented with bootstrapped data, was also used (Ruscio and Roche 2012). In addition, the *psych* package (Revelle 2015) includes criteria such as Velicer’s Minimum Average Partial (MAP) (Velicer 1976), Schwarz’s Bayesian Information Criterion (BIC) (Schwarz 1978) and the sample size adjusted variant (SSABIC) (Sclove 1987) to select the number of factors to retain. These showed little agreement regarding the number of factors to retain, but suggest overall a large number of factors (i.e. > 6) were optimal. The parallel analysis like previous uses (Stewart and Austin 2009) indicated an eight factor solution, as did BIC. The comparison data approach suggested a 12 factor approach, as did Kaiser’s criterion and similar to SSABIC (11). However, the plot of these factors indicated a levelling off beyond eight factors, and these explained little additional variance in the data. Three methods: Velicer’s MAP (3), Optimal Coordinates (6) and Acceleration Factor (1) suggested a small number of factors should be retained. Consequently, a principal axis exploratory factor analysis retaining eight factors was conducted on the AQ data, using an orthogonal (varimax) rotation to produce independent factors in order to construct composite indicators. The output of the factor analysis is displayed in Table 1. Item 30 was subtracted from subscale scores on factor 2 due to its negative loading.

**Table 1:** Factor loadings for the varimax rotated eight factor model

ITEM	F1	F2	F3	F4	F5	F6	F7	F8
38	0.74							
44	0.73							0.23
17	0.73							
11	0.72							
47	0.68					0.21		
22	0.57	0.36						
15	0.55			0.23				
26	0.50	0.41						
13	0.48							0.25
46	0.39	0.35				0.32		−0.25
1	0.28							
24	0.25							0.2
45		0.54		0.32				
33	0.25	0.45						
20		0.43						
35		0.43						
42		0.4						
39		0.38						
7		0.36						0.26
30		−0.26					0.22	
12			0.66					
23			0.6					
5			0.49			0.21		
6			0.47				0.24	
16		0.28	0.41					
4		0.25	0.4					
19		0.23	0.35				0.26	
27	0.22			0.61				
36	0.3			0.6				
31	0.21	0.22		0.55				
32	0.23			0.36				
37				0.35				
10	0.31			0.34	0.21			
48	0.28			0.33				
50	0.23				0.54			
8				0.21	0.49			
40					0.47			
14					0.46			
3					0.41			
25	0.25					0.47		
34	0.35					0.43		
2		0.29				0.4		
43		0.21	0.26			0.34		
28				0.26		0.28		
29							0.55	
49							0.52	
9							0.27	0.2
18		0.25						0.36
41		0.2	0.32					0.32
21								0.26

Only loadings > 0.2 included

Taxometric analysis has three key assumptions. The first is that putative indicators show substantial differences between a proposed taxon and non-taxon (or complement), quantified using the standardised between-groups effect size Cohen’s *d* that ought to exceed 1.25 (Meehl 1995). Indicators entered into taxometric analyses should show little *nuisance covariance*, meaning they are relatively uncorrelated (mean *r* < 0.3) among taxon and non-taxon cases (Ruscio et al. 2006). Finally both the overall dataset and the proposed taxon should contain enough cases. A minimum sample size of 300 is recommended for taxometric analysis, and taxon base rate should be at least 5 % of the total sample and preferably 10 % (Walters and Ruscio 2009).

Initial checks of indicator validity revealed that multiple indicators showed substantial nuisance covariance or insufficient separation between taxon and complement (see Supplementary Materials). As many items on the problematic indicators showed substantial cross loading onto other factors, these were merged into four composite indicators (Table 2) that met the prerequisite assumptions. After merging indicators seven items were not included in the taxometric analysis. Indicators did not appear to meet criteria for substantial skew or kurtosis (West et al. 1995).

**Table 2:** Items included in the taxometric analysis using four indicators

Indicator 1	Indicator 2	Indicator 3	Indicator 4
38	45	12	25
44	33	23	34
17	20	5	2
11	35	6	43
47	42	16	28
22	39	4	
15	7	19	
26	30*	41	
13	18		
46			
1			
24			
27			
36			
31			
32			
37			
10			
48			
50			
8			

Please note for indicator 2 that the score for item 30 was subtracted from the sum of the remaining items

### Analytic Procedure

Taxometric analysis was conducted using an *R* script developed by Ruscio (2013). The data was compared against 100 samples of bootstrapped data with idealised categorical and dimensional latent structure. These are used to calculate a comparison curve fit index (CCFI), which ranges between 0 and 1. A CCFI > 0.5 suggests the categorical data better fits the observed data, < 0.5 the dimensional data, and indices between 0.6 and 0.4 should be treated as ambiguous.

Cases were assigned to taxon and complement based on a cut-off of 32 or more (Woodbury-Smith et al. 2005; Baron-Cohen et al. 2001). Four taxometric analyses were conducted; MAMBAC (Mean Above, Minus Below A Cut) (Meehl and Yonce 1994), MAXCOV (Maximum Covariance) (Meehl 1973), MAXEIG (Maximum Eigenvalue) (Waller and Meehl 1998) and L-Mode (Latent Mode) (Waller and Meehl 1998) Factor Analysis. MAMBAC (**M**ean **A**bove **M**inus **B**elow **A C**ut) searches for an optimal cutting score by looking at the mean difference between scores above and below a series of sliding cuts along the input variable. In MAXCOV (**Max**imum **Cov**ariance) the input is sorted into subsamples that will vary in the proportion of taxon and complement members; in the presence of a taxon covariance occurs with a mixture of taxon and non-taxon members, and should be maximal when the subsample is equally comprised of the two. MAXEIG (**Max**imum **Eig**envalue) instead computes the first eigenvalue from a modified covariance matrix for all of the output indicators from a number of overlapping windows. L-Mode (**L**atent **Mode**) Factor Analysis plots the weighted least squares factor scores from a single latent factor to examine whether the distribution is bimodal (Walters et al. 2010).

MAMBAC was conducted with 50 evenly spaced cuts beginning 25 cases from each extreme iterating through each input/output combination. MAXEIG analysis was conducted with each indicator serving as input and the remaining as output, producing a number of curves equal to the number of indicators. MAXCOV analysis was conducted using triplets of input/output/output variables. In both cases, the input variable was portioned into 25 windows with an overlap of 0.9.

## Results

All four analyses supported the presence of a taxon, with an examination of the comparison curves revealing a categorical structure was a much better fit of the data. With the exception of the L-Mode Factor Analysis (Fig. 5), the dimensional comparison data was a strikingly poor fit of the observed data. In all cases the CCFI’s were greater than 0.6, supporting a categorical model. The results from each analysis are discussed in detail below.

The base rates suggested slight differences between the different types of taxometric analysis. The MAXCOV, MAXEIG and L-Mode analyses suggested a taxon base rate of around 0.15, or 15 % of the sample across all measures. This is similar to the proportion of cases that are greater or equal to 32, the cut-off suggested in the literature for non-clinical samples (Baron-Cohen et al. 2001). However, the base rates from the MAMBAC analysis were closely aligned with the proportion of cases greater than or equal to 26, a cut-off hypothesized to correspond to the presence of what was referred to as higher functioning ASD in previous research using clinical samples (Woodbury-Smith et al. 2005).

### MAMBAC Analysis

**Fig. 2:**
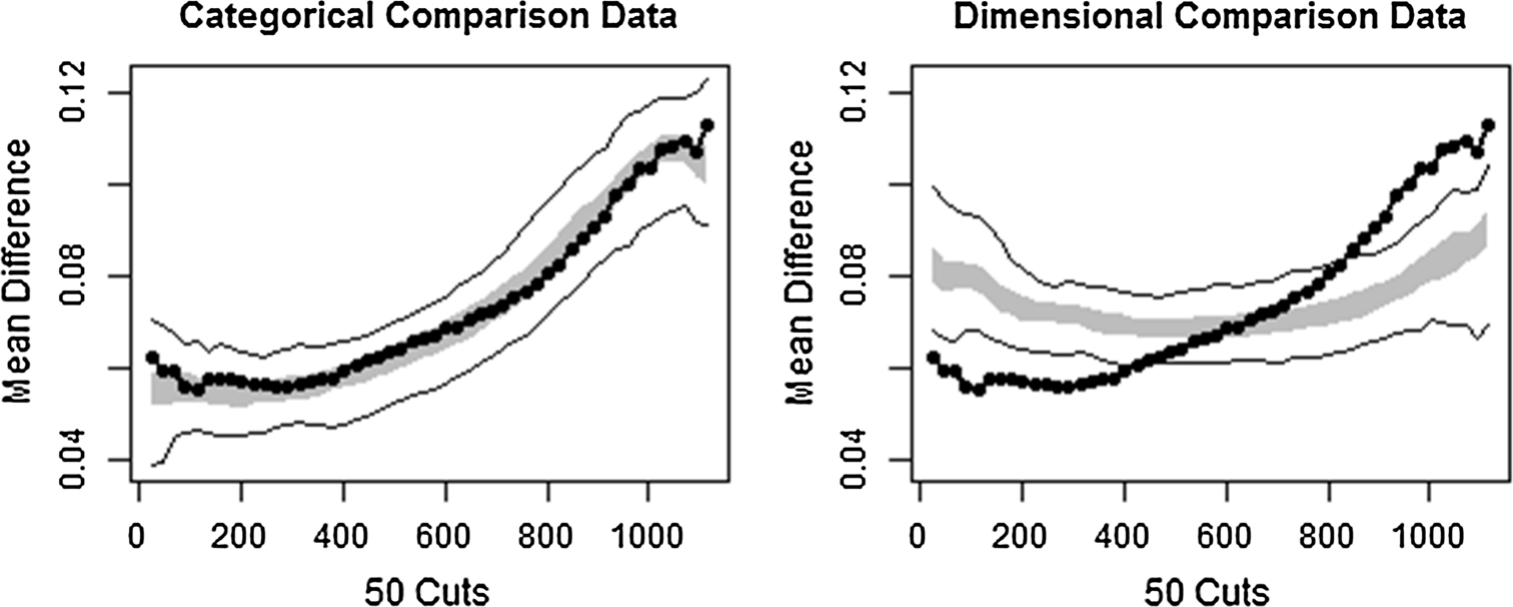
Comparison data from mean above minus below a cut (MAMBAC) analysis (CCFI = 0.862). The *grey band* represents the medium 50 % of the data points from the bootstrapped data that have the same distributional statistics and distribution as the observed sample, but with idealised latent structures. The *solid black* lines represent the total range of the bootstrapped comparison data. The *dotted black line* is the averaged taxometric curve

Figure 2 shows the averaged MAMBAC curve compared against comparison categorical and dimensional data. The graph shows that the categorical comparison and observed data are a close fit whereas the dimensional data is a poor fit outside of the centre of the plot. A CCFI of 0.862 supports this interpretation. The base rate estimate for the averaged curve is 0.357, closely corresponding to the proportion of the sample meeting the cut-off of low severity ASD identified in previous analyses of AQ data (Woodbury-Smith et al. 2005). The mean base rate across indicators was 0.362 (*S.D.* = 0.077).

### MAXCOV/MAXEIG Analysis

**Fig. 3:**
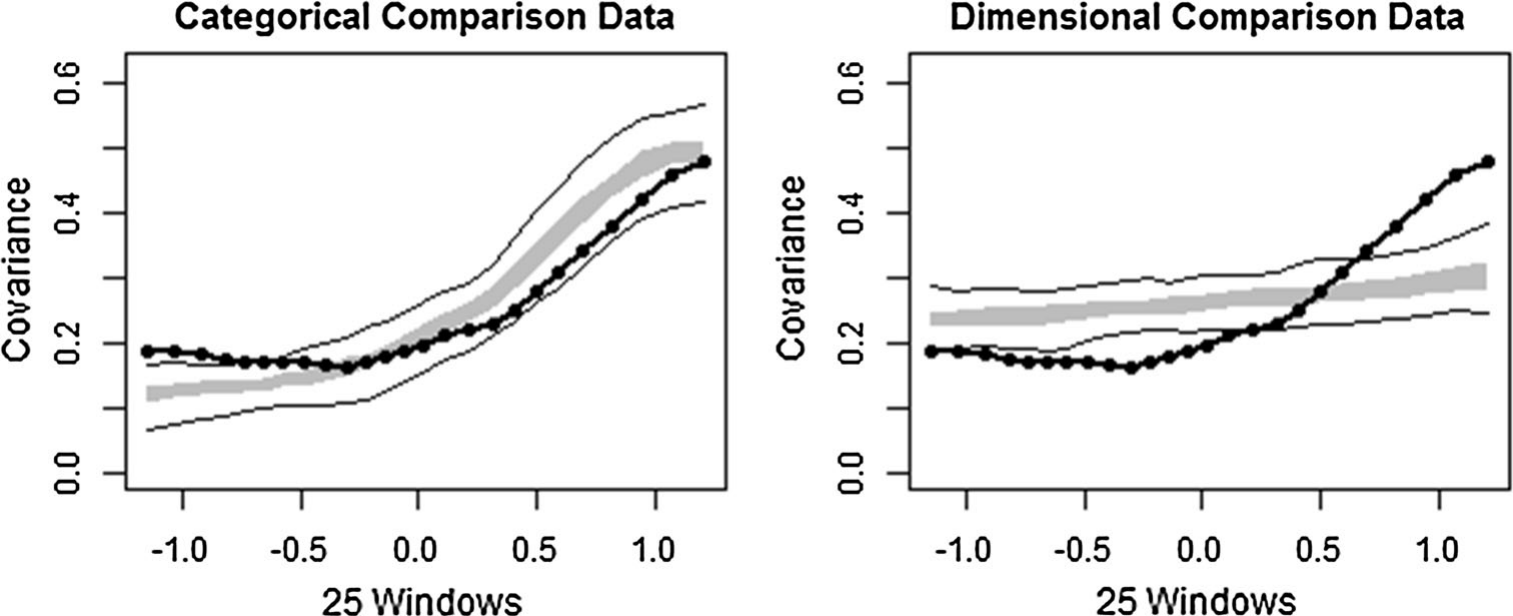
Comparison data from maximum covariance (MAXCOV) analysis (CCFI = 0.669). The *grey band* represents the medium 50 % of the data points from bootstrapped data that have the same distributional statistics and distribution as the observed sample, but with idealised latent structures. The *solid black lines* represent the total range of the bootstrapped comparison data. The *dotted black line* is the averaged taxometric curve

**Fig. 4:**
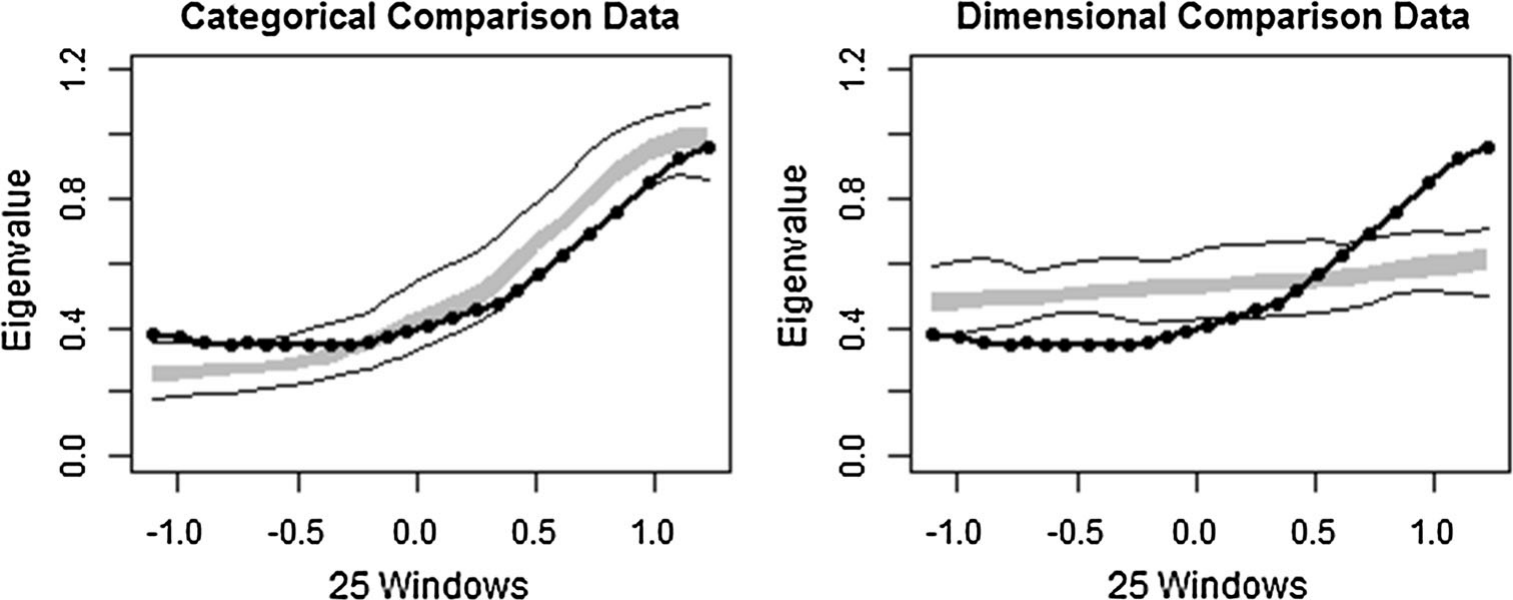
Comparison data from maximum eigenvalue (MAXEIG) analysis (CCFI = 0.68). The *grey band* represents the medium 50 % of the data points from bootstrapped data that have the same distributional statistics and distribution as the observed sample, but with idealised latent structures. The *solid black lines* represent the total range of the bootstrapped comparison data. The *dotted black line* is the averaged taxometric curve

The comparison curves were very similar for both analyses (Figs. 3 and 4) demonstrating that the categorical comparison data is an adequate fit of the comparison data. The dimensional comparison data appears to show only weak correspondence with the observed data. The CCFI for the MAXCOV analysis was 0.669, supporting a categorical interpretation. The base rates differed from the MAMBAC analysis; the base rate estimate for the average MAXCOV curve was 0.165, corresponding with the proportion of the sample that scored ≥32. The average base rate across the indicators was 0.168 (*S.D.* = 0.034).

The same trend emerged for the MAXEIG findings; the observed data was a reasonable fit of the categorical but not the dimensional comparison data, a CCFI of 0.68 again indicates strong support for a categorical interpretation, and a base rate of 0.166 on the average curve supports the presence of a taxon broadly corresponding to a cut-off of 32. The average base rate across the curves was 0.168 (*S.D.* = 0.029).

### L-Mode Factor Analysis

**Fig. 5:**
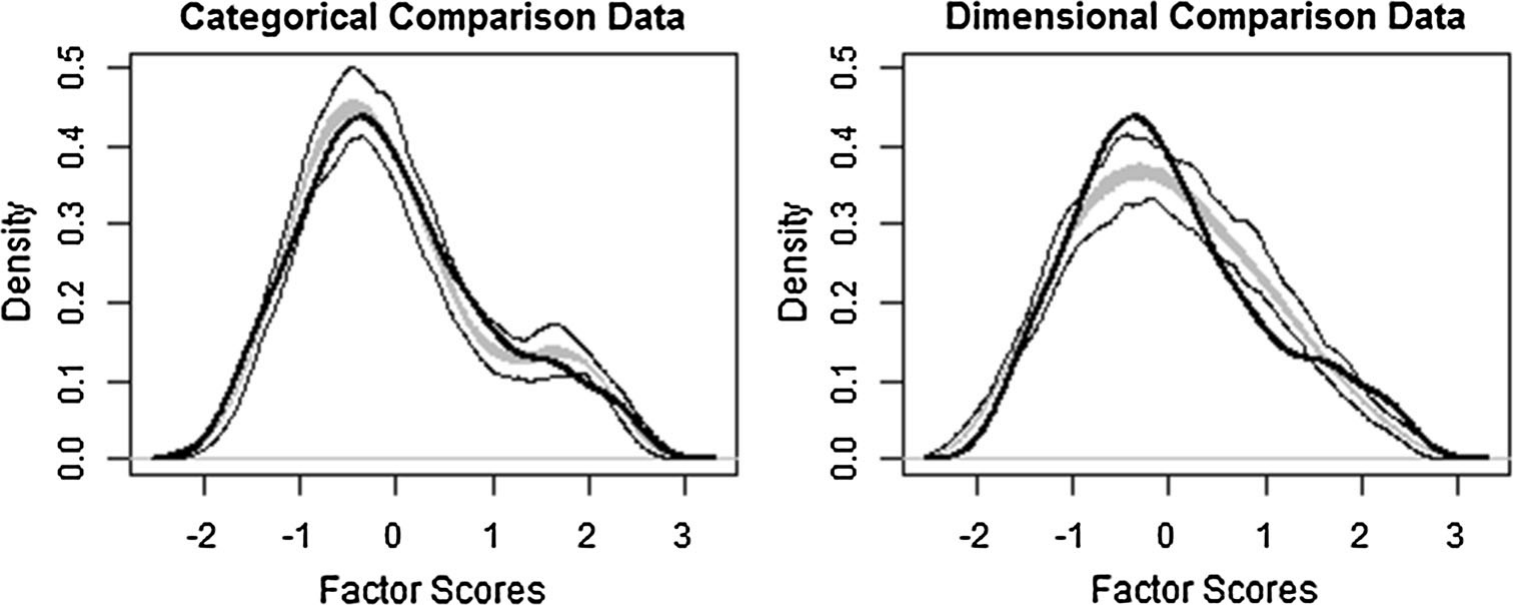
Comparison data from Latent Mode (L-Mode) Factor Analysis (CCFI = 0.775). The *grey band* represents the medium 50 % of the data points from bootstrapped samples that have the same distributional statistics and distribution as the observed sample, but with idealised latent structures. The *solid black lines* represent the total range of the bootstrapped comparison data. The *dotted black line* is the averaged taxometric curve

The L-Mode comparison curve (Fig. 5) shows that while the simulated categorical data is a better fit of the data than the dimensional data, there is not a clear bimodal distribution in the observed data. There is a secondary peak at the right side of the distribution potentially indicative of a small base-rate taxon, and this was an example of where the categorical comparison data was a better fit than the dimensional data; a small base rate population may be disguised by the tail of the distribution of a more prevalent population (Murphy 1964). A similar secondary peak was also found in pooled non-clinical data (Ruzich et al. 2015). The CCFI for the L-Mode Factor Analysis was 0.775, suggesting the data were a better fit of a categorical model. The estimated base rate was 0.12, in line with MAXCOV and MAXEIG results.

## Discussion

Four taxometric analyses were conducted on indicators constructed from the AQ. The results of these analyses fail to support the assertion that the AQ is measuring quantitative differences in ATs. Instead, the evidence appears to support a qualitative difference, although the different analyses did not decisively agree on where this categorical boundary lay. This perhaps indicates the presence of multiple latent classes. The results strongly supported the presence of a qualitative difference at a score of 32, based on pre-specifying a potential taxonic group and from the base rates of the taxometric curves. MAMBAC analyses provided mixed support for a cut-off at a base rate almost identical to a score of 26, a boundary between ASD and non-ASD in ATs previously identified in the literature, albeit under certain conditions (Woodbury-Smith et al. 2005). Our findings strongly suggest that the latent structure of the construct measured by the AQ is categorical in nature, and there appears to be an AT taxon.

One limitation of taxometric analysis is that it cannot provide a definitive answer as to the number of taxa present in a latent construct. The divergence in estimates between base rates, and their correspondence to two established cut off points of differing ASD severity suggest that further analysis is required. Although all of the analyses found that the AQ measured at least one qualitative difference, taxometric analysis is relatively weak at identifying the number of latent classes present in a dataset (McGrath and Walters 2012). These findings suggest that the AQ may contain multiple latent classes and as such identifying the categorical latent structure of the AQ using another analytic technique, latent class analysis, is warranted. The next section reports the findings of a latent class analysis of AQ data.

## Study 2a: Latent Class Analysis of AQ Data

Latent class analysis (LCA) is a form of latent variable modelling that derives mutually exclusive subtypes or categories of case from responses to categorical indicators. Parameters of the latent class model, namely the prevalence of latent class membership and item-response probabilities for each indicator, are estimated using a maximum likelihood approach (Collins and Lanza 2010). LCA has been frequently contrasted with factor analysis as both are designed to uncover an underlying structure from a series of measured variables. However, factor analysis assumes that the latent variable is dimensional, and tends to assume that the measured variables are continuous as well. We conducted a latent class analysis on the AQ items, predicting there would be at least two or three latent classes on the basis of the taxometric analysis in Study 1.

## Method

### Sample

The sample was the same as reported in Study 1.

### Analytic Procedure

LCA was conducted on the fifty dichotomized AQ items. Latent class models between one and nine latent classes were estimated. The analysis was conducted using MPlus v. 6.1.2 (Muthén and Muthén 1998–2011). Competing models were compared using a range of indices of model fit. These include the classification accuracy or entropy of the model, Akaike Information Criterion (Akaike 1974), BIC (Schwarz 1978) and SSABIC (Sclove 1987), and a series of bootstrapped and non-nested likelihood ratio tests to compare alternative latent class models. Previous studies (Nylund et al. 2007) have indicated that BIC is the most robust measure of model fit in latent class analysis, particularly with larger sample sizes, and so greater weight was assigned to this index.

## Results

Indices of model fit were compared across the latent class models. BIC indices for 1 through 9 latent classes (Table 3) revealed the six-class model was the best fit of the data. As previous studies have indicated, AIC tended to over fit the data, identifying highly complex models. An examination of the distributions of AQ scores (Supplementary Materials) reveals a latent class similar to the taxonic group identified in study 1 (Class 6); this group shows a very high AQ score, almost exclusively exceeding 32. Three intermediate groups (Classes 1, 2 and 5) and two groups appearing to endorse few ATs (Classes 3 and 4) were also observed. The means and standard deviations for these are reported in Table 4.

**Table 3:** Indices of model fit for LCA of AQ items

	AIC	BIC	SSABIC	Entropy	LMR-LRT	VLMR-LRT	BLRT
1-class	72,989.58	73,241.48	73,082.67	–	–	–	–
2-class	66,259.47	66,768.30	66,447.49	**0.93**	<0.001	<0.001	<0.001
3-class	64,619.39	65,385.16	64,902.36	0.92	<0.001	<0.001	<0.001
4-class	64,076.67	65,099.37	64,454.58	0.87	0.04	0.04	<0.001
5-class	63,626.73	64,906.36	64,099.58	0.87	**0.02**	**0.02**	<0.001
6-class	63,347.01	**64,883.57**	63,914.80	0.87	0.16	0.16	<0.001
7-class	63,136.56	64,930.06	63,799.29	0.86	0.63	0.63	<0.001
8-class	62,984.12	65,034.55	63,741.80	0.87	0.79	0.79	<0.001
9-class	**62,845.15**	65,152.51	**63,697.76**	0.87	0.75	0.75	**<0.001**

Values in bold identify the number of classes a statistical test indicates is the best fitting model

For AIC, BIC and SSABIC, this is the lowest reported information criterion. For entropy this is the highest reported statistic. For LRT’s this is the final model in which the *p* value is significant

**Table 4:** Means and standard deviations of AQ scores for each of the subgroups identified by LCA

Class	Mean	Standard deviation
1 (14.66 %)	24.89	4.18
2 (13.87 %)	23.39	4.58
3 (24.58 %)	15.65	3.20
4 (21.07 %)	11.51	3.90
5 (10.45 %)	24.37	4.59
6 (15.37 %)	37.88	4.38

The posterior probability of class membership for each class was used to determine the pseudo-class membership for each case to help interpret the latent classes (Table 4 and Supplementary Materials). The term ‘endorsement’ is used in regard to items where the behaviour indicative of the presence of an AT is affirmed. In a minority of the bivariate residuals between indicators there was evidence of residual covariance, which might indicate that local independence was violated. An overall test of local independence could not be computed because of the size of the contingency table. The characteristics of each latent class are reported below.

### Taxonic Group

This group made up 15.37 % of the analysed sample. An examination of the response probabilities for this group reveals that 43 of the 50 AQ items had more than a 50 % probability of being endorsed. Five of the seven items that were unlikely to be frequently endorsed by cases in this class were items on indicators excluded from the taxometric analysis in Study 1 due to insufficient between-groups separation. Items 21, 3 and 9 showed particularly low probabilities of endorsement (<33 %). One item (11) had a conditional response probability of 100 %, and items 17, 26, 38, 46, 22, 4 and 12 exceeded 95 %. This latent class showed comparatively better classification accuracy compared to the other latent classes, further indicating the presence of a latent taxon.

### Intermediate Latent Classes

There were three latent classes with almost identical levels of AT severity. However, the conditional response probabilities for the AQ items revealed differences between classes. Interpretation began by comparing the estimated endorsement probabilities of each item across the indicators entered into the taxometric analysis reported in Study 1 (Table 2). This revealed that the estimated parameters for Class 1 showed lower probabilities of endorsement for items comprising the first taxometric indicator compared to other latent classes with similar AQ scores; for each item bar one (24) on the first factor in Table 1, the lowest probability was located in Class 1. Although the estimated probabilities were comparatively lower, these tended to range between 0.2 and 0.4, whereas for the other classes of similar AQ severity this often exceeded 0.7. The first taxometric indicator comprises many of the same items as the *Social Skills* factor in a previously identified three factor solution (Austin 2005) that strongly correlated with the three components of the classic autistic triad (Wing and Gould 1979). Classes 2 and 5 showed similar levels of endorsement on this factor. Cases in Class 2 responded less to items cross loading onto the fourth and fifth factors reported in Table 1. A similar pattern emerged for the second factor in Table 1 and Class 2. This second factor comprised similar items again to Austin’s (2005) three factor finding, this time ‘*Communication/Mindreading*’, as well as the second indicator entered into the taxometric analysis. Class 1 tended to show higher rates of endorsement for these items, and Class 5 appeared to show a mixed pattern of responding, alternating between strong and moderate endorsement probabilities for these items. Again the same pattern emerged with the third factor (and taxometric indicator), with Class 5 showing lower probabilities of endorsement for this factor, and Classes 1 and 2 demonstrating moderate and high probabilities of endorsement. This factor also closely corresponded to one of Austin’s (2005) three factors, this time the ‘*Details/Patterns’* factor. Latent classes showed only very minor differences on the fourth taxometric indicator, which measured items related to repetitive behaviours in the context of routines.

### Low Severity Latent Classes

Two classes were also uncovered that demonstrated very low AQ scores. One of these groups (Class 4) showed very low endorsement of almost every AQ item. Only four items had a conditional response probability exceeding 50 % in this subgroup: 14, 2, 46 and 30. The third class showed slightly higher severity, but included a number of items that had a higher probability of endorsement. An examination of the items that with a higher response probability against previous factor analyses of AQ items revealed that this was systematic; with the exception of one item (30, which negatively loaded onto the second factor) all of the items strongly endorsed loaded onto the third factor (‘*Details/Patterns’*). This indicator also includes items related to very deeply held interests, systemising behaviours, and the list of items appear broadly congruent with the non-social autistic traits identified by systemising-based accounts in the literature (Baron-Cohen et al. 2009).

### Gender

We further tested whether class membership differed between subgroups. A multinomial logistic regression was estimated on the most likely latent class individuals were assigned to, using gender as an indicator (coded female = 0, male = 1). Class 4 (low scores, high systemising) was chosen as the reference class as this had a female to male ratio similar to the overall sample. This revealed that three groups had a different gender distribution: Class 1 (moderate severity, lower probability of endorsing social skills difficulties, higher probabilities on details/patterns and communication/mindreading indicators) had a greater log odds of being male (*b* = 0.516, *S.E.* = 0.199, *p* = 0.01, 95 % CI 0.125–0.906), Class 4 (low probability on all indicators) had a greater log odds of being female (*b* = −0.927, *S. E.* = 0.206, *p* = < 0.001, 95 % CI –1.331–−0.523) and Class 6 (taxonic group) had a greater log odds of being male (*b* = 0.814, *S.E.* = 0.199, *p* < 0.001, 95 % CI 0.423–1.205). Classes 2 (*b* = 0.048, *S.E.* = 0.206, *p* = 0.814, 95 % CI −0.355–0.452) and 5 (*b* = 0.355, *S.E.* = 0.225, *p* = 0.137, 95 % CI −0.106–0.775) showed no significant difference. The variance explained by gender is very small (McFadden’s *R* ^*2*^ = 0.02), but significant (*G* ^*2*^ = 77.66, *p* < 0.001). Males and females in the taxonic group showed no differences in their responding to the different taxometric indicators (between-subjects *t* tests *p* > 0.05).

## Study 2b: Latent Profile Analysis

We estimated latent profile models (LPA) using the taxometric indicators in Study 1, as it appeared some indicators might have violated LCA’s local independence assumption. Latent profile models of between one and nine classes were estimated. The indicators met the local independence assumption. The LRT’s supported a five-class model, and BIC a seven-class model. Examination of BIC indices revealed only very minor differences between five and nine class models (Table 5). Regardless, these models showed similar characteristics; all of the models identified a latent class of individuals showing very high response probability for almost every item comprising around 16 % (5 and 6 class) or 12 % (7 + class) of the sample. These correspond with the base rates identified by MAXCOV/MAXEIG and L-Mode taxometric procedures. Additionally there was a group comprising around 30–35 % of the sample that had a systematically higher probability of endorsing systemising related items. These also identify one or two (8 or 9 class models) with low probability of response for the vast majority of items. Where these models differ is in the number of intermediate subtypes identified. A five class model identified two intermediate classes, one displaying higher scores on social skills and systemising indicators and the second showed relatively high scores (but lower than the taxon group) on all four of the indicators. The six class model (Table 6) identified three classes similar to the LCA, and subsequent models tended to identify further groups along similar lines, albeit with continually smaller subtype samples. In light of the findings of the LCA, we focus on the six-class model.

**Table 5:** Indices of model fit for latent profile analysis of AQ items

	AIC	BIC	SSABIC	Entropy	LMR-LRT	VLMR-LRT	BLRT
1-class	21,461.55	21,501.85	21,476.44	–	–	–	–
2-class	20,403.52	20,469.02	20,427.72	0.85	<0.001	<0.001	<0.001
3-class	20,187.64	20,278.32	20,221.15	0.82	<0.001	<0.001	<0.001
4-class	20,102.41	20,218.28	20,145.22	0.79	0.03	0.03	<0.001
5-class	20,031.55	20,172.61	20,083.67	0.74	0.03	0.03	<0.001
6-class	19,988.01	20,154.26	20,049.44	0.75	0.34	0.33	<0.001
7-class	19,947.96	20,139.40	20,018.70	0.75	0.69	0.69	<0.001
8-class	19,924.33	20,140.96	20,004.38	0.75	0.07	0.07	<0.001
9-class	19,901.84	20,143.66	19,991.20	0.74	0.44	0.45	<0.001

**Table 6:** Results of the estimated model for each of the indicators entered into the LPA analysis (standard errors in brackets)

	Indicator 1	Indicator 2	Indicator 3	Indicator 4
Class 1 (20.38 %)	3.61 (0.22)	0.53 (0.11)	2.16 (0.18)	1.70 (0.13)
Class 2 (32.72 %)	4.63 (0.44)	1.10 (0.12)	5.29 (0.16)	2.28 (0.10)
Class 3 (12.14 %)	12.92 (0.66)	1.66 (0.22)	3.04 (0.50)	2.58 (0.21)
Class 4 (4.56 %)	9.44 (1.27)	5.09 (1.05)	6.43 (0.25)	2.37 (0.52)
Class 5 (14.36 %)	11.59 (2.06)	1.68 (0.43)	5.93 (0.37)	3.15 (0.24)
Class 6 (15.84 %)	17.29 (0.25)	5.34 (0.34)	6.82 (0.14)	4.28 (0.10)

Because indicator 2 included an item that negatively loaded onto this indicator, scores ranged from −1 to 8

Like the LCA, the estimated six-class latent profile model identifies a latent class (Class 6) that resembled the taxonic group, showing very high scores on all four of the indicators, and showed a similar pseudo-class membership rate to the LCA of 16.4 %. In addition there was a latent class (Class 1) that scored low on all four indicators, and a low scoring class (Class 2) comprising around 30 % of the sample that displayed strong endorsement of the indicator probing attention to detail, patterns and very strongly held interests (or systemising). In both 5 and 6 class models this included a larger membership than the LCA. There were again three intermediate classes. One of these displayed higher scores on the first (social skills difficulties) indicator (Class 3), another showing very high scores on the second and third indicators (Class 4) and a third strongly endorsing the first and third indicators (Class 5), much in the same manner as the LCA. Again with the exception of the taxonic subtype there were relatively little differences between subtypes on the fourth indicator, measuring repetitive and routine related behaviours.

Comparing the most likely class membership between LPA and LCA confirms the similarities observed. The low severity and taxonic LPA groups comprise most of the same respondents as the corresponding LCA groups. Where the analyses seemed to diverge more was regarding the intermediate latent classes. The first class from the LCA (showing low endorsement of social skills, high endorsement of systematising behaviours and communication/mindreading difficulties) made up the majority of cases in the fourth LPA class, but only around a fifth of cases were assigned to this group. The remainder were predominantly assigned to the low severity, high systematising group (LPA class 2) and the fifth LPA subtype (high social skills and communication/mindreading difficulties). The second LCA class (low detail, high social, moderate attentional) was mostly assigned to the fifth LPA class but about 25 % of cases were assigned to the second and third LPA subtypes respectively. The fifth LCA class was overwhelmingly assigned to the third LPA class. The similarity between class assignments was relatively high (72.26 %), with the majority of differences emerging between LCA class 1 and 2, which comprised two-thirds of the cases in which LCA and LPA disagreed. For the low severity and taxon class, the agreement rate between analyses was 89.37 %.

## Discussion

Latent class analyses of AQ data revealed the presence of six latent classes. There was a clear ASD taxon defined by high AQ severity and a high probability of endorsing most AQ items, which had higher classification accuracy than the other latent classes. The proportion of the sample belonging to this class was similar to three taxometric procedures reported in Study 1. There were three distinct intermediate groups, displaying a higher probability of engaging in behaviours comprising one or two previously discovered dimensions that appear to correspond to the autistic triad: social skills, communication/mindreading and details/patterns (or systemising) (Austin 2005). Two low severity classes were also discovered, one unlikely to endorse more than a few AQ items, the second only likely to endorse items measuring attention to detail or seeing patterns in events, items probing repetitive interests or behaviours that are linked with a systemising account of non-social autistic traits. However, it was unclear whether the items as entered met the local independence assumption of LCA. An LPA, conducted to overcome this limitation, revealed a similar pattern of results. The LPA failed to conclusively support a specific latent class model, but a six-class LPA produced a very similar structure to the LCA and class membership for the two analyses tended to converge. The indicator scores from the LPA reflected the same pattern as the estimated latent class model for individual items.

## General Discussion

The Autism Quotient is assumed to measure an underlying continuum that ranges from minimal difficulties with functions such as social skills, communication and flexibility of thought or repetitive restricted behaviours, to individuals who meet or are likely to meet the diagnostic criteria for ASD. Our findings indicate that the latent structure of the most prevalent screen of ATs are best characterised as containing a distinct latent class endorsing all three components of the autistic triad with further classes endorsing different components respectively. The presence of a latent class was notably consistent across three different analytic approaches, two of which also identified three classes showing similar levels of AT but endorsing behaviours symptomatic of different parts of the autistic triad. The analyses demonstrate there are distinct subtypes within the AQ, indicative of a mixed or categorical structure. This finding is similar to others who, upon examining the latent distribution of ATs across populations with and without ASD, report evidence of a taxon in high AT severity (Frazier et al. 2012, 2010).

Studies adopting a dimensional approach to the AQ have suggested that very high scores (> 3 SD’s from the mean) should be considered as belonging to a ‘narrow autism phenotype’. Individuals in this phenotype are anticipated to either have an ASD diagnosis or likely meet the criteria but have not sought diagnosis (Wheelwright et al. 2010). Although defined in reference to the extreme end of a continuum of AT, scores for this group are similar to the identified taxon in this analysis, suggesting that these individuals instead form a distinct latent class discontinuous from other cases on the AQ. Because this group appears to be highly robust between analyses and similar to the AQ cut-off indicative of clinically significant levels of autistic traits, this supports the idea of using the AQ as a screening tool for ASD. However, a study comparing various assessments of autistic symptoms in ASD individuals found no relationship between AQ scores and standard clinical measures of ASD (i.e. ADI-R) or adaptive behaviour (i.e. VABS) (Bishop and Seltzer 2012). Furthermore, another study using item response modelling of AQ data indicated that the assessment does not capture very high levels of AT in a mixed ASD/non-ASD sample (Murray et al. 2015a). This raises qualifications concerning whether the construct (and taxon) measured by the AQ necessarily generalises to ASD in the manner a dimensional explanation might expect. Although the reported analyses suggest that ATs measured by the AQ do not measure a latent continuum, psychometric analyses of a wider range of assessments that also measure ATs would be beneficial. There has been comparatively less research on the relationship between the AQ and other assessments apart from the Social Responsiveness Scale (Armstrong and Iarocci 2013), particularly in ASD respondents. Although there is a taxon of respondents endorsing all three components of the autistic triad, additional studies with a clinical sample or follow-up assessment might identify what this would translate into. This is especially important in light of the changes to the diagnostic criteria for ASD in the DSM-5, where it is conceptualised as a dyad of impairments and there is the introduction of a separate diagnosis of Social Communication Disorder.

The second consideration is whether AQ data should be interpreted along subscales. In addition to querying the continuity of AQ scores, these analyses suggest that looking at the degree to which different types or domains of ATs are endorsed may be more informative than total AQ score, particularly in respondents that might fall into the broad autism phenotype. The original AQ report includes subscale scores but these analyses are auxiliary and their use in the literature has been sparse, primarily because there is little consensus on the factor structure of the AQ. Two analyses (EFA and LCA) suggest the presence of three factors that appear to map onto the autistic triad of impairments. It is worth noting that several analyses (Austin 2005; Hurst et al. 2007; Palmer et al. 2015) have found a similar factor structure in nonclinical samples. Outside of cases that might fall into the broad autism phenotype, two classes of respondents scoring around the non-clinical mean AQ score were identified (Ruzich et al. 2015), one of which showed low endorsement of all behaviours and one systematically endorsing RRB or systemising behaviours. Previous analyses have found that the attention to detail factor (which covers many repetitive, systemising behaviours) behaved separately to other subscales when modelling a general factor alongside the pre-specified five subscales of the AQ (Murray et al. 2015b). This led to the suggestion that these items should be decoupled from computing a total AQ score. The findings of this analysis do not substantially deviate from this recommendation in individuals outside the apparent taxon.

The latent class analyses revealed a number of distinct subtypes showing similar overall severity, but with systematic differences emerging in the type of autistic traits endorsed. The sub threshold, intermediate severity classes appear to present themselves in particular components of the autistic triad proposed by Wing and Gould (1979). Scores for these three groups encompasses a similar albeit slightly broader range on the AQ to a construct that has previously been referred to as the broader autism phenotype (Wheelwright et al. 2010), in which individuals endorse a considerable number of autistic traits. This refers to individuals scoring 1–2 standard deviations above the mean, which in previous studies was an AQ score between 23 and 28, on the basis of a non-clinical sample. The concept that these separate components may show fractionation, albeit primarily within individuals with ASD, has previously been explored (Happé and Ronald 2008; Brunsdon and Happé 2014), suggesting that there may be distinct causal mechanisms for different components of ASD. The findings of the LCA/LPA are consistent with this line of research, identifying distinct subgroups of individuals endorsing aspects of the triad rather than a single intermediate severity group (which might have supported a dimensional account). However it should be noted that these groups showed the greatest divergence between analyses, although both LCA and LPA revealed the same broad structure for this band of severity.

Previous analyses have identified social communication/interaction difficulties and restricted repetitive behaviours as separate components in ASD alongside an ASD/non-ASD latent category (Frazier et al. 2012). We found strong evidence to support the presence of both of these factors plus an additional third factor relating to communication and mindreading/theory of mind deficits. Many of the items on the third indicator, which probed attention to detail and obsessive interests, congruent with the category of restricted and repetitive behaviour, are often highlighted in the concept of systemising (Baron-Cohen et al. 2009). Further research should be undertaken to understand why there is consistent and compelling evidence to suggest there is a subtype of individuals that shows very little likelihood of endorsing autistic traits or behaviours other than those related to restricted repetitive behaviours/systemising. This is of considerable interest in regard to this sample as in both LCA and LPA this was the modal group, comprising between 25 and 33 % of the total sample.

The presence of multiple intermediate latent classes provides a fairly clear explanation as to why additional taxometric analyses might fail to identify the presence of low or moderate severity taxa in the AQ dataset, namely because the items that comprised the indicators systematically differed between three classes of almost equal AQ score. This is a finding that might be important when taxometrics is used to try and test for the presence of more than two latent classes in a dataset (Ruscio and Ruscio 2002). This also indicates the sort of scenario where an iterative taxometric approach may be less informative, and that LCA should be conducted instead.

The highest scores on the AQ and those likely to endorse significant social and communication difficulties were more likely to be male, whereas females tend to be more prominent in the group that scored lower across all subcomponents of the AQ. This fits with the general finding that ASD and high levels of AT are more frequently diagnosed in males than in females (Newschaffer et al. 2007). One might question whether females with autism show different symptoms that are not being captured by the AQ (Gould and Ashton-Smith 2011). For example, sensory issues are underrepresented in the AQ and therefore the measure is less sensitive to detecting sensory atypicalities in ASD individuals. Some evidence has shown that females with ASD report more lifetime sensory symptoms (Lai et al. 2011), indicating that these may be a more ‘female’ phenotype for ASD which is not fully represented in the current version of the AQ.

One possibility is that the use of mixture modelling may provide further clarification on how to conceptualise AT. Analysis of autism data have suggested that hybrid models appear more parsimonious than latent class models (Frazier et al. 2012), and that mixture modelling tends to improve model fit. While this is unlikely to make any difference to the taxonic class identified by taxometric analysis and LCA/LPA, for the sub-threshold classes such an analysis may prove informative. The data suggests that a three-factor model mixed with a latent class model would be an optimal fit of the data. In the context of the methods used, it would also be interesting to compare the base rate of the taxon-like class when a mixture model is fitted. Though taxometric analysis is one of the most prominent methods of testing between dimensional and categorical latent models, this has not been without controversy. A mixture modelling approach may be more likely to identify valid latent classes (Lubke and Tueller 2010) although findings in this area are divergent (Cleland et al. 2000).

Overall, these findings have implications to consider for the practical use of the AQ given its widespread popularity. The primary observation is that there is reason to query the utility of treating total AQ score as a quantitative variable, particularly in samples that span the cutoff the AQ developers suggested is indicative of clinically significant levels of AT (Baron-Cohen et al. 2001). In many studies AQ score is used as a quantitative variable in order to correlate, predict or otherwise differentiate one group from another on a common scale. Analyses of abbreviated versions of the AQ across individuals with and without ASD suggested caution ought to be taken comparing AQ score across groups as threshold invariance was not observed (Murray et al. 2014). A qualification with the findings of the present analysis is that the previous literature has raised concerns about differences between clinical and general samples (Ruzich et al. 2015). As such, further analyses using a clinical sample would be highly beneficial in order to replicate and extend the current findings. Similarly, comparing the latent class structure against data that is taken from a nationally representative sample of the general population would be beneficial. While these findings are theoretically coherent, it remains to be seen whether they translate to similar results in a group that is representative of the entire population. The sample shows a similar distribution of AQ scores to other online samples but respondents scored higher than other non-clinical samples (Ruzich et al. 2015). In addition responses are heavily sampled from the student population, but this is typical of many AQ samples including the original validation study (77 % student sample). The online sample included recruitment from groups that may or may not show clinical levels of AT—the proportion of respondents comprising this group are not known as ASD diagnosis was not queried. Furthermore the AQ is designed to measure AT in a population with a normal IQ. Many individuals with ASD show impairments in this domain, and IQ between ASD/non-ASD cases represents a taxonic distinction (Ingram et al. 2008). Measures of intelligence were not collected as part of this study. The AQ assumes respondents do not have learning or linguistic difficulties, and further analyses should replicate the findings of the present analysis accounting for these variables. Similarly, information about level of education, employment or primary language was not collected which would be beneficial to account for. Further research may compliment existing work that has looked at a shortened version of the AQ (Kuenssberg et al. 2014). As the results of these analyses suggest a taxon is present in AT data, the findings from these or other analyses with clinical or nationally representative samples might be beneficial at further optimising a shortened version of the AQ. The latent class model identifies the probability of each AQ item being endorsed, and could be used to identify a reduced set of items that identify taxon members efficiently.

To conclude, we conducted a taxometric analysis of AQ data that supported the presence of a latent taxon. Examination of taxon base rates suggested that multiple latent classes might be present in the data. Consequently latent class and latent profile models were subsequently estimated. These indicated a six-class model was the best fit of the data. Sub-threshold classes showed fractionation of facets of ATs along different components of the autistic triad. The results strongly suggest that analyses that account for this may be more appropriate than treating AQ scores as a continuous variable.

## Electronic supplementary material

Below is the link to the electronic supplementary material.

Supplementary material 1 (DOCX 55 kb)

## Footnotes

1 Taxometric analyses were conducted on the data with different rules for truncating missing data to examine whether these affected the results. Comparisons between MAMBAC, MAXEIG and L-Mode analyses where only the three extreme cases were removed (CCFI = 0.862 MAMBAC, 0.68 MAXEIG, 0.75 L-Mode), cases where more than 20 % of data was missing were removed (four cases removed, CCFI = 0.866, 0.684, 0.79), and 10 % (12 cases removed, CCFI = 0.858, 0.681, 0.788), revealed only very minor differences between the outcomes, with increasingly omitting data tending to even further support a categorical model for L-Mode and MAXEIG analyses, and demonstrating very little difference between MAMBAC analyses.
