# Supplementary material for: The Latent Structure of Autistic Traits: A Taxometric, Latent Class and Latent Profile Analysis of the Adult Autism Spectrum Quotient
Source: J Autism Dev Disord. 2016 Sep 12;46(12):3712–28. doi: 10.1007/s10803-016-2897-z (PMC5110592; doi:10.1007/s10803-016-2897-z)
Supplement: Supplementary file 1 — Supplementary material 1 (DOCX 55 kb) [file 10803_2016_2897_MOESM1_ESM.docx]

Missing data

If data are not ‘missing completely at random’ (MCAR), use of complete-case analysis can lead to biased results ([Little and Rubin 2002](#_ENREF_4)). Homoscedasiticy of the data was tested using the TestMCARNormality function ([Jamshidian and Jalal 2010](#_ENREF_2)), which is part of the MissMech package in R ([Jamshidian et al. 2014](#_ENREF_3)). The test of homoscedasiticy was rejected, indicating that the data violated the MCAR assumption. When data are not MCAR, it is recommended that the missing values are imputed to avoid the issues stated above ([Graham 2009](#_ENREF_1)). We used the R package missForest ([Stekhoven 2013](#_ENREF_5); [Stekhoven and Bühlmann 2012](#_ENREF_6)) to impute the missing data. Although multiple methods have been developed in order to deal with missing data - including single/multiple imputation, multivariate imputation by chained equations and nearest neighbour estimation - missForest has been demonstrated to introduce the least imputation error and has the smallest prediction difference from actual non-imputed values ([Waljee et al. 2013](#_ENREF_7)). Random forest imputation is able to effectively impute values for cases which have up to 30% missing data, meaning that more cases can be included in the final analysis.

Indicator construction

Table S1 outlines the skew, kurtosis and between-groups effect size statistics for each of the indicators. Table 4 reports the correlations between the indicators, revealing that there was substantial nuisance covariance for Indicators 3 and 4 with other variables in the non-taxon group. Indicator 4 was very highly correlated with Indicator 1 among non-taxon members. However, it is not surprising that the majority of items on Indicator 4 cross-loaded (> .25) onto Indicator 1. Factor analyses conducted based on other criteria (e.g. Velicer’s MAP) found that with a smaller number of factors that the items that comprised Indicators 1 and 4 tended to load onto the same factor. Consequently, these indicators were merged. Indicators 5, 7 and 8 did not demonstrate sufficient between groups separation to justify their continued inclusion. Furthermore an examination of the factor loadings revealed that many of the items on these indicators loaded weakly onto the factors, and showed some cross loading onto other factors. Three of the six items on indicator 5 also loaded onto indicators 4 or 1. Consequently, these were merged into Indicator 1. Two of the three items that comprised indicator 8 also loaded onto Indicator 2; these items were instead included in Indicator 2. The remaining items that showed low loading onto the composite indicators, a total of seven of the fifty items that comprise the AQ, were not included for further taxometric analysis. It should be noted excluding items and merging indicators did not substantially change the findings of the taxometric analysis; even when poor indicators were included the analyses were in the same direction as the results reported below.

Table S1. Skew, kurtosis and between-groups effect size statistics for indicators derived from exploratory factor analysis, and the merged indicators entered into the taxometric analysis.

| Indicator | Skew | Kurtosis | Cohen’s *d* |
| --- | --- | --- | --- |
| *All indicators* | | | |
| 1 | 0.27 | -1.26 | 1.99 |
| 2 | 0.72 | 0.30 | 2.21 |
| 3 | -0.48 | -0.65 | 1.17 |
| 4 | 0.53 | -0.82 | 2.10 |
| 5 | 0.73 | -0.35 | 1.09 |
| 6 | -0.02 | -0.99 | 1.47 |
| 7 | 0.29 | -0.97 | 0.15 |
| 8 | 0.67 | -0.38 | 1.02 |
|  |  |  |  |
| *Merged Indicators* | | | |
| 1 | 0.42 | -0.97 | 2.33 |
| 2 | 0.73 | -0.20 | 2.18 |
| 3 | -0.33 | -0.73 | 1.29 |
| 4 | 0.02 | -0.99 | 1.47 |

Table S2. Correlations for the putative AQ indicators derived from the exploratory factor analysis using clinical cutoff (> 32).

| Full Sample (N = 1139) | | | | | | | | |
| --- | --- | --- | --- | --- | --- | --- | --- | --- |
|  | 1 | 2 | 3 | 4 | 5 | 6 | 7 | 8 |
| 1 | - |  |  |  |  |  |  |  |
| 2 | 0.50 | - |  |  |  |  |  |  |
| 3 | 0.34 | 0.39 | - |  |  |  |  |  |
| 4 | 0.61 | 0.59 | 0.24 | - |  |  |  |  |
| 5 | 0.32 | 0.32 | 0.05 | 0.34 | - |  |  |  |
| 6 | 0.44 | 0.38 | 0.31 | 0.40 | 0.27 | - |  |  |
| 7 | -0.04 | 0.01 | 0.20 | -0.08 | -0.02 | 0.05 | - |  |
| 8 | 0.21 | 0.38 | 0.39 | 0.21 | 0.11 | 0.21 | 0.09 | - |
| Taxon Group (N = 188) | | | | | | | | |
|  | 1 | 2 | 3 | 4 | 5 | 6 | 7 | 8 |
| 1 | - |  |  |  |  |  |  |  |
| 2 | -0.03 | - |  |  |  |  |  |  |
| 3 | 0.07 | 0.11 | - |  |  |  |  |  |
| 4 | 0.21 | 0.23 | -0.19 | - |  |  |  |  |
| 5 | -0.07 | 0.04 | -0.13 | 0.08 | - |  |  |  |
| 6 | 0.22 | 0.09 | 0.20 | 0.19 | -0.02 | - |  |  |
| 7 | -0.16 | -0.06 | 0.13 | -0.13 | 0.03 | 0.01 | - |  |
| 8 | 0.04 | 0.33 | 0.18 | 0.05 | 0.07 | 0.02 | 0.09 | - |
| Complement Group (N = 951) | | | | | | | | |
|  | 1 | 2 | 3 | 4 | 5 | 6 | 7 | 8 |
| 1 | - |  |  |  |  |  |  |  |
| 2 | 0.22 | - |  |  |  |  |  |  |
| 3 | 0.15 | 0.20 | - |  |  |  |  |  |
| 4 | 0.41 | 0.35 | 0.001 | - |  |  |  |  |
| 5 | 0.16 | 0.13 | -0.12 | 0.17 | - |  |  |  |
| 6 | 0.23 | 0.11 | 0.14 | 0.14 | 0.14 | - |  |  |
| 7 | -0.09 | -0.03 | 0.21 | -0.15 | -0.06 | 0.03 | - |  |
| 8 | 0.01 | 0.19 | 0.31 | -0.02 | -0.05 | 0.05 | 0.08 | - |

Table S3. Covariance and nuisance covariance between merged AQ indicators.

| Full sample |  |  |  |  |
| --- | --- | --- | --- | --- |
|  | 1 | 2 | 3 | 4 |
| 1 | - |  |  |  |
| 2 | 0.57 | - |  |  |
| 3 | 0.37 | 0.43 | - |  |
| 4 | 0.48 | 0.37 | 0.32 | - |
| Taxon |  |  |  |  |
| 1 | - |  |  |  |
| 2 | 0.12 | - |  |  |
| 3 | -0.09 | 0.23 | - |  |
| 4 | 0.22 | 0.10 | 0.18 | - |
| Complement |  |  |  |  |
| 1 | - |  |  |  |
| 2 | 0.28 | - |  |  |
| 3 | 0.13 | 0.24 | - |  |
| 4 | 0.24 | 0.10 | 0.14 | - |

Table S4. Distribution of scores for each of the latent classes based on the most likely class membership for each case.

| Score | 1 | 2 | 3 | 4 | 5 | 6 |
| --- | --- | --- | --- | --- | --- | --- |
| 2 | 0 | 0 | 0 | 1 | 0 | 0 |
| 4 | 0 | 0 | 0 | 3 | 0 | 0 |
| 5 | 0 | 0 | 1 | 6 | 0 | 0 |
| 6 | 0 | 0 | 0 | 8 | 0 | 0 |
| 7 | 0 | 0 | 2 | 27 | 0 | 0 |
| 8 | 0 | 0 | 1 | 17 | 0 | 0 |
| 9 | 0 | 0 | 6 | 17 | 0 | 0 |
| 10 | 0 | 0 | 6 | 26 | 1 | 0 |
| 11 | 0 | 0 | 9 | 23 | 0 | 0 |
| 12 | 0 | 0 | 11 | 20 | 0 | 0 |
| 13 | 0 | 1 | 33 | 15 | 0 | 0 |
| 14 | 0 | 2 | 38 | 16 | 1 | 0 |
| 15 | 0 | 3 | 30 | 16 | 1 | 0 |
| 16 | 2 | 6 | 28 | 17 | 1 | 0 |
| 17 | 2 | 3 | 31 | 10 | 4 | 0 |
| 18 | 5 | 8 | 33 | 9 | 3 | 0 |
| 19 | 6 | 12 | 21 | 6 | 2 | 0 |
| 20 | 5 | 15 | 13 | 1 | 14 | 0 |
| 21 | 17 | 9 | 5 | 2 | 10 | 0 |
| 22 | 16 | 12 | 10 | 0 | 7 | 0 |
| 23 | 15 | 10 | 1 | 0 | 5 | 0 |
| 24 | 17 | 6 | 1 | 0 | 9 | 0 |
| 25 | 15 | 16 | 0 | 0 | 7 | 0 |
| 26 | 16 | 8 | 0 | 0 | 12 | 0 |
| 27 | 6 | 14 | 0 | 0 | 12 | 0 |
| 28 | 8 | 8 | 0 | 0 | 11 | 1 |
| 29 | 8 | 12 | 0 | 0 | 5 | 1 |
| 30 | 7 | 6 | 0 | 0 | 3 | 2 |
| 31 | 9 | 3 | 0 | 0 | 3 | 8 |
| 32 | 6 | 2 | 0 | 0 | 4 | 10 |
| 33 | 5 | 0 | 0 | 0 | 2 | 11 |
| 34 | 1 | 2 | 0 | 0 | 1 | 11 |
| 35 | 1 | 0 | 0 | 0 | 1 | 11 |
| 36 | 0 | 0 | 0 | 0 | 0 | 12 |
| 37 | 0 | 0 | 0 | 0 | 0 | 16 |
| 38 | 0 | 0 | 0 | 0 | 0 | 21 |
| 39 | 0 | 0 | 0 | 0 | 0 | 12 |
| 40 | 0 | 0 | 0 | 0 | 0 | 10 |
| 41 | 0 | 0 | 0 | 0 | 0 | 16 |
| 42 | 0 | 0 | 0 | 0 | 0 | 9 |
| 43 | 0 | 0 | 0 | 0 | 0 | 4 |
| 44 | 0 | 0 | 0 | 0 | 0 | 6 |
| 45 | 0 | 0 | 0 | 0 | 0 | 1 |
| 46 | 0 | 0 | 0 | 0 | 0 | 10 |
| 47 | 0 | 0 | 0 | 0 | 0 | 1 |
| 49 | 0 | 0 | 0 | 0 | 0 | 2 |

Table S5. Response probabilities and standard errors for each of the AQ items, for each of the six latent classes.

| Item | 1 (14.96%) | 2 (13.96%) | 3 (24.46%) | 4 (20.84%) | 5 (10.47%) | 6 (15.32%) |
| --- | --- | --- | --- | --- | --- | --- |
| 1 | 0.43 (0.05) | 0.67 (0.08) | 0.32 (0.04) | 0.20 (0.03) | 0.58 (0.07) | 0.73 (0.04) |
| 2 | 0.70 (0.05) | 0.54 (0.06) | 0.56 (0.04) | 0.52 (0.04) | 0.58 (0.07) | 0.93 (0.02) |
| 3 | 0.20 (0.04) | 0.10 (0.04) | 0.09 (0.02) | 0.22 (0.04) | 0.40 (0.07) | 0.30 (0.04) |
| 4 | 0.85 (0.04) | 0.71 (0.05) | 0.70 (0.04) | 0.40 (0.05) | 0.67 (0.06) | 0.96 (0.02) |
| 5 | 0.75 (0.05) | 0.70 (0.05) | 0.76 (0.04) | 0.34 (0.05) | 0.52 (0.07) | 0.91 (0.03) |
| 6 | 0.70 (0.06) | 0.58 (0.06) | 0.70 (0.06) | 0.24 (0.04) | 0.27 (0.07) | 0.77 (0.04) |
| 7 | 0.42 (0.06) | 0.21 (0.05) | 0.15 (0.03) | 0.12 (0.03) | 0.19 (0.06) | 0.75 (0.04) |
| 8 | 0.29 (0.05) | 0.16 (0.04) | 0.10 (0.02) | 0.18 (0.03) | 0.43 (0.07) | 0.46 (0.04) |
| 9 | 0.43 (0.06) | 0.27 (0.05) | 0.27 (0.04) | 0.08 (0.02) | 0.07 (0.04) | 0.33 (0.04) |
| 10 | 0.56 (0.06) | 0.48 (0.05) | 0.22 (0.03) | 0.29 (0.04) | 0.84 (0.05) | 0.84 (0.03) |
| 11 | 0.50 (0.06) | 0.88 (0.05) | 0.11 (0.04) | 0.10 (0.03) | 0.91 (0.04) | 1.00 (0.00) |
| 12 | 0.80 (0.06) | 0.96 (0.03) | 0.95 (0.03) | 0.49 (0.06) | 0.44 (0.09) | 0.95 (0.02) |
| 13 | 0.35 (0.05) | 0.63 (0.09) | 0.22 (0.04) | 0.08 (0.03) | 0.55 (0.08) | 0.88 (0.03) |
| 14 | 0.45 (0.05) | 0.37 (0.06) | 0.25 (0.04) | 0.52 (0.06) | 0.63 (0.07) | 0.53 (0.04) |
| 15 | 0.33 (0.06) | 0.62 (0.07) | 0.24 (0.03) | 0.08 (0.02) | 0.61 (0.07) | 0.91 (0.02) |
| 16 | 0.80 (0.05) | 0.54 (0.06) | 0.61 (0.04) | 0.31 (0.06) | 0.46 (0.07) | 0.90 (0.03) |
| 17 | 0.25 (0.06) | 0.84 (0.10) | 0.14 (0.03) | 0.10 (0.03) | 0.76 (0.07) | 0.98 (0.02) |
| 18 | 0.41 (0.05) | 0.21 (0.04) | 0.39 (0.04) | 0.24 (0.04) | 0.15 (0.05) | 0.54 (0.05) |
| 19 | 0.78 (0.05) | 0.50 (0.07) | 0.48 (0.05) | 0.14 (0.04) | 0.29 (0.07) | 0.71 (0.04) |
| 20 | 0.37 (0.06) | 0.04 (0.02) | 0.07 (0.02) | 0.11 (0.03) | 0.20 (0.06) | 0.57 (0.04) |
| 21 | 0.31 (0.05) | 0.12 (0.04) | 0.17 (0.03) | 0.15 (0.03) | 0.12 (0.04) | 0.30 (0.04) |
| 22 | 0.45 (0.06) | 0.71 (0.06) | 0.12 (0.03) | 0.13 (0.03) | 0.75 (0.07) | 0.96 (0.02) |
| 23 | 0.78 (0.06) | 0.79 (0.06) | 0.82 (0.05) | 0.20 (0.05) | 0.37 (0.08) | 0.95 (0.02) |
| 24 | 0.48 (0.05) | 0.45 (0.05) | 0.38 (0.04) | 0.24 (0.04) | 0.47 (0.06) | 0.74 (0.04) |
| 25 | 0.58 (0.05) | 0.61 (0.06) | 0.40 (0.04) | 0.28 (0.04) | 0.56 (0.06) | 0.92 (0.03) |

| Item | 1 (14.96%) | 2 (13.96%) | 3 (24.46%) | 4 (20.84%) | 5 (10.47%) | 6 (15.32%) |
| --- | --- | --- | --- | --- | --- | --- |
| 26 | 0.72 (0.05) | 0.80 (0.05) | 0.17 (0.04) | 0.23 (0.04) | 0.84 (0.05) | 0.97 (0.01) |
| 27 | 0.56 (0.07) | 0.16 (0.05) | 0.10 (0.03) | 0.18 (0.03) | 0.67 (0.07) | 0.93 (0.02) |
| 28 | 0.48 (0.05) | 0.53 (0.06) | 0.48 (0.04) | 0.28 (0.04) | 0.57 (0.06) | 0.82 (0.04) |
| 29 | 0.51 (0.05) | 0.53 (0.05) | 0.58 (0.04) | 0.45 (0.04) | 0.31 (0.06) | 0.48 (0.04) |
| 30 | 0.50 (0.06) | 0.71 (0.05) | 0.78 (0.03) | 0.63 (0.04) | 0.37 (0.06) | 0.47 (0.04) |
| 31 | 0.29 (0.05) | 0.07 (0.04) | 0.07 (0.02) | 0.10 (0.02) | 0.34 (0.06) | 0.76 (0.04) |
| 32 | 0.49 (0.05) | 0.38 (0.05) | 0.24 (0.03) | 0.23 (0.04) | 0.60 (0.06) | 0.77 (0.04) |
| 33 | 0.45 (0.05) | 0.34 (0.06) | 0.12 (0.03) | 0.08 (0.03) | 0.39 (0.07) | 0.80 (0.03) |
| 34 | 0.32 (0.05) | 0.38 (0.05) | 0.11 (0.02) | 0.18 (0.03) | 0.38 (0.06) | 0.78 (0.04) |
| 35 | 0.42 (0.07) | 0.18 (0.04) | 0.12 (0.03) | 0.20 (0.04) | 0.27 (0.06) | 0.58 (0.04) |
| 36 | 0.40 (0.07) | 0.15 (0.05) | 0.07 (0.02) | 0.09 (0.02) | 0.64 (0.07) | 0.88 (0.03) |
| 37 | 0.55 (0.06) | 0.45 (0.05) | 0.25 (0.04) | 0.34 (0.05) | 0.56 (0.06) | 0.81 (0.03) |
| 38 | 0.46 (0.07) | 0.93 (0.04) | 0.09 (0.03) | 0.11 (0.03) | 0.89 (0.04) | 0.97 (0.01) |
| 39 | 0.56 (0.06) | 0.32 (0.06) | 0.33 (0.04) | 0.13 (0.03) | 0.25 (0.06) | 0.74 (0.04) |
| 40 | 0.24 (0.04) | 0.24 (0.05) | 0.17 (0.03) | 0.18 (0.04) | 0.33 (0.07) | 0.56 (0.04) |
| 41 | 0.55 (0.06) | 0.42 (0.06) | 0.30 (0.04) | 0.03 (0.02) | 0.17 (0.05) | 0.73 (0.04) |
| 42 | 0.57 (0.06) | 0.21 (0.04) | 0.29 (0.04) | 0.24 (0.04) | 0.37 (0.07) | 0.79 (0.04) |
| 43 | 0.73 (0.05) | 0.72 (0.05) | 0.62 (0.04) | 0.48 (0.04) | 0.52 (0.07) | 0.92 (0.02) |
| 44 | 0.10 (0.03) | 0.47 (0.10) | 0.01 (0.01) | 0.01 (0.01) | 0.53 (0.09) | 0.81 (0.04) |
| 45 | 0.61 (0.08) | 0.21 (0.06) | 0.13 (0.03) | 0.19 (0.04) | 0.57 (0.08) | 0.93 (0.02) |
| 46 | 0.76 (0.05) | 0.88 (0.04) | 0.50 (0.04) | 0.57 (0.04) | 0.89 (0.03) | 0.97 (0.02) |
| 47 | 0.16 (0.04) | 0.54 (0.10) | 0.06 (0.02) | 0.05 (0.02) | 0.63 (0.08) | 0.83 (0.03) |
| 48 | 0.37 (0.05) | 0.29 (0.05) | 0.10 (0.02) | 0.16 (0.03) | 0.63 (0.06) | 0.66 (0.04) |
| 49 | 0.40 (0.05) | 0.46 (0.05) | 0.53 (0.04) | 0.42 (0.04) | 0.31 (0.06) | 0.35 (0.04) |
| 50 | 0.43 (0.05) | 0.36 (0.06) | 0.20 (0.04) | 0.21 (0.04) | 0.64 (0.06) | 0.68 (0.04) |

REFERENCES

Graham, J. W. (2009). Missing Data Analysis: Making It Work in the Real World. *Annual Review of Psychology, 60*(1), 549-576, doi:doi:10.1146/annurev.psych.58.110405.085530.

Jamshidian, M., & Jalal, S. (2010). Tests of Homoscedasticity, Normality, and Missing Completely at Random for Incomplete Multivariate Data. *Psychometrika, 75*(4), 649-674, doi:10.1007/s11336-010-9175-3.

Jamshidian, M., Jalal, S. J., & Jansen, C. (2014). Missmech: an R package for testing homoscedasticity, multivariate normality, and missing completely at random (MCAR). *Journal of Statistical Software, 56*(6).

Little, R. J. A., & Rubin, D. B. (2002). *Statistical analysis with missing data* (2nd ed.). New York: John Wiley & Sons.

Stekhoven, D. J. (2013). missForest: Nonparametric Missing Value Imputation using Random Forest. R package version 1.4.

Stekhoven, D. J., & Bühlmann, P. (2012). MissForest—non-parametric missing value imputation for mixed-type data. *Bioinformatics, 28*(1), 112-118, doi:10.1093/bioinformatics/btr597.

Waljee, A. K., Mukherjee, A., Singal, A. G., Zhang, Y., Warren, J., Balis, U., et al. (2013). Comparison of imputation methods for missing laboratory data in medicine. *BMJ Open, 3*(8), doi:10.1136/bmjopen-2013-002847.
